# Cross-cultural validation of the birth memories and recall questionnaire: a cross-sectional study among Chinese postpartum women

**DOI:** 10.7717/peerj.20814

**Published:** 2026-02-26

**Authors:** Yuling Zhu, Jie Hua, Simei Zhou, Qian Zhou, Shurong Huang, Wenzhi Cai, Ling Chen

**Affiliations:** 1 Department of Nursing, Shenzhen Hospital, Southern Medical University, Shenzhen, Guangdong, China; 2 School of Nursing, Southern Medical University, Guangzhou, Guangdong, China; 3 Department of Obstetrics, Shenzhen LongHua Maternity & Child Healthcare Hospital, Shenzhen, Guangdong, China; 4 Department of Community Health Service Center, The People’s Hospital of Baoan Shenzhen, Shenzhen, Guangdong, China; 5 Department of Obstetrics and Gynaecology, Shenzhen Hospital, Southern Medical University, Shenzhen, Guangdong, China

**Keywords:** Birth memory, Postpartum depression, Childbirth-related post-traumatic stress disorder, Cross-cultural validation

## Abstract

**Background:**

Birth memory is closely linked to the psychological well-being of postpartum women, highlighting the importance of its accurate assessment. However, no specialized and validated instrument is currently available in China to evaluate birth memory. This study aimed to cross-culturally adapt the Birth Memories and Recall Questionnaire for Chinese postpartum women and validate its psychometric properties.

**Methods:**

The Birth Memories and Recall Questionnaire was translated into Chinese and culturally adapted following the Beaton model. Content validity was assessed by an expert panel. A convenience sample of 494 primiparous women, aged 18 to 49 years, who had undergone vaginal childbirth and had an infant aged 0 to 12 months, was recruited from three tertiary-level public hospitals in China. Construct validity was tested using confirmatory factor analysis, along with assessments of convergent validity, discriminant validity, and measurement invariance. Known-group validity was assessed by comparing birth memory characteristics between women with and without probable postpartum depression (PPD) and childbirth-related post-traumatic stress disorder (CB-PTSD). Reliability was established through internal consistency and test-retest reliability.

**Results:**

The Chinese version of the Birth Memories and Recall Questionnaire exhibited satisfactory content validity, with a scale-level content validity index of 0.95. Confirmatory factor analysis showed an acceptable model fit and supported the six-factor structure of the original questionnaire. The questionnaire demonstrated good convergent and discriminant validity, and it supported measurement invariance across PPD symptom subgroups. Women with probable PPD or CB-PTSD reported more emotional memory, reliving, centrality of memory, and involuntary recall. Women with probable PPD also reported less coherence, while those with probable CB-PTSD reported more sensory memory. The Chinese version of the questionnaire demonstrated high internal consistency, with an overall Cronbach’s alpha of 0.83 and McDonald’s omega of 0.81. Test-retest reliability was confirmed with an intra-class correlation coefficient of 0.73.

**Conclusions:**

The Chinese version of the Birth Memories and Recall Questionnaire is a valid and reliable tool for assessing the birth memory characteristics of postpartum women, which will be valuable in future research and clinical practice.

## Introduction

Childbirth is often regarded as a positive and life-changing event for women. However, for some, it can also be distressing or even traumatic (Alcorn et al., 2010) potentially increasing their vulnerability to postpartum psychological disorders, such as depression and post-traumatic stress disorder (PTSD). Studies indicate that up to 14.0% of women experience postpartum depression (PPD) (Liu, Wang & Wang, 2022), while 4.7% are affected by childbirth-related PTSD (CB-PTSD) during the postpartum period (Heyne et al., 2022). As major public health concerns (Wisner, Chambers & Sit, 2006; Bener, Gerber & Sheikh, 2012), PPD and CB-PTSD not only significantly impair maternal health (Ayers, Eagle & Waring, 2006; Slomian et al., 2019) but also disrupt the mother-infant relationship (Rados et al., 2020; Frankham, Thorsteinsson & Bartik, 2023), hinder child developmental outcomes (Garthus-Niegel et al., 2017; Sridhar, Kishore & Chandra, 2025), and impose substantial economic burdens on families and society(Luca et al., 2020; Bauer et al., 2022). Consequently, identifying modifiable risk factors, particularly those related to childbirth event itself that affect maternal mental health, and developing targeted prevention and intervention strategies to reduce the incidence and slow the clinical progression of postpartum psychological disorders are of significant importance.

Birth memory refers to the unique information encoded and retained in long-term memory related to the childbirth experience, which can be recalled as needed. Prior research has demonstrated that birth memory influences maternal perception of traumatic childbirth (Altuntuğ, Kiyak & Ege, 2023; Yilmaz & Kiliç, 2024), overall functioning (Ozcalik & Aksoy, 2024), and adaptation to parenthood (Karakoç, Bekmezci & Meram, 2022; Çark & Çankaya, 2024). The characteristics of birth memory are closely associated with postpartum mood regulation and play a significant role in the onset and progression of postpartum psychological disorders (Briddon, Isaac & Slade, 2015; Hughes et al., 2020; Hutchinson & Cassidy, 2022), particularly PPD and CB-PTSD (Foley et al., 2014; Thiel et al., 2021). Therefore, accurate assessment of birth memory characteristics is crucial for identifying women with negative or traumatic memories and for developing targeted interventions to prevent and control the onset and progression of postpartum psychological disorders. However, to date, no instrument has been specifically developed or psychometrically validated to measure birth memory characteristics in Chinese populations. This gap significantly limits research on the relationship between birth memories and postpartum psychological disorders among Chinese women and hinders the development of culturally appropriate interventions.

The Birth Memory and Recall Questionnaire (BirthMARQ), developed by Foley et al. (2014), is currently the only validated instrument specifically designed to assess birth memory characteristics in postpartum women. It consists of 21 items across six dimensions: Emotional Memory, Reliving, Centrality of Memory, Sensory Memory, Recall, and Coherence. Emotional Memory evaluates the valence of emotions experienced during childbirth and during the recall of the birth; Reliving measures how much the birth experience is re-experienced; Centrality of Memory assesses the extent to which the birth memory has been integrated into the mother’s self-identity and life story; Sensory Memory measures how well women remember details of smells, tastes, sounds, and touches from childbirth; Recall assesses the frequency of spontaneous, voluntary, and prompted recall of the birth; and Coherence evaluates the degree to which the memory is perceived as complete and well-organized or fragmented (Foley et al., 2014). These comprehensive and interrelated dimensions enable the questionnaire to capture unique aspects of the birth experience and postpartum adaptation, providing a more tailored and sensitive assessment of birth memory characteristics than general autobiographical memory measures such as the Memory Characteristics Questionnaire (Johnson et al., 1988), the Autobiographical Memories Questionnaire (Rubin, Schrauf & Greenberg, 2003), and the Memory Experiences Questionnaire (Sutin & Robins, 2007).

To date, the BirthMARQ has demonstrated favorable psychometric properties across two cultural contexts. In its original development study (Foley et al., 2014), the construct validity, known-group validity, and internal consistency were tested in an online sample of 523 postpartum women within one year after childbirth in the United Kingdom. Construct validity was supported by principal components analysis, which extracted six factors with all item loadings above 0.30. Known-group validity was confirmed by significant dimensional score differences between women with and without probable PPD or CB-PTSD. Internal consistency was acceptable, with Cronbach’s alpha values ranging from 0.74 to 0.84 across subscales (Foley et al., 2014). In 2021, the BirthMARQ was translated into Turkish and validated in a sample of 387 postpartum women within one year after childbirth (Topkara & Çağan, 2021). In addition to confirming the good reliability and validity reported in the original study, the Turkish version further evaluated the questionnaire’s content validity. A scale-level content validity index of 0.95 was obtained, indicating that the questionnaire also had good content validity (Topkara & Çağan, 2021). However, the applicability and psychometric properties of the BirthMARQ in Chinese populations remain unexplored and warrant further investigation. Therefore, this study aimed to cross-culturally adapt the 21-item BirthMARQ for Chinese postpartum women and validate its psychometric properties.

## Methods

### Study design

This research is an instrument adaptation and multicenter cross-sectional validation study conducted with a structured process that involves two phases. In Phase I, the BirthMARQ underwent translation and cross-cultural adaptation in accordance with the Beaton model (Beaton et al., 2000), which encompasses six key stages: initial translation, synthesis, back translation, expert committee review, pretesting, and submission of documentation to the developers. In Phase II, the psychometric properties of the Chinese version of the BirthMARQ were evaluated. This study was reported in line with the Strengthening the Reporting of Observational Studies in Epidemiology (STROBE) checklist (Table S1) (Vandenbroucke et al., 2007).

### Phase I: translation and cross-cultural adaptation

Ayers was contacted by email, and authorization to adapt the BirthMARQ for Chinese postpartum women was obtained. The Beaton model was applied to guide the translation and cross-cultural adaptation of the BirthMARQ (Beaton et al., 2000). In Stage 1, two native bilingual speakers independently translated the BirthMARQ into Chinese, producing versions T1 and T2. One translator was a nursing postgraduate student who was aware of the concepts examined in the questionnaire, while the other was a nursing postdoctoral researcher who was unfamiliar with the specific constructs measured by the instrument and had completed her doctoral studies in Hong Kong, China. In Stage 2, the project leader and translators examined the initial translations (T1, T2) against the original instrument and produced a common version (T-12). In Stage 3, a back-translation process was performed, during which two bilingual translators, who had no exposure to the original English version, were unfamiliar with the study objectives, and were working in English-speaking countries, independently translated the T-12 version back into English (BT1, BT2). In Stage 4, a meeting was convened involving all translators and the research team to compare and discuss the translated and back-translated versions alongside the original questionnaire, resolve any discrepancies, and reach consensus on the wording of the prefinal Chinese version. In Stage 5, 30 primiparous women who were not involved in the main study were invited to fill in the questionnaire, and assess the clarity and comprehension of the instructions, items, and options. The results showed that all items could be understood. In Stage 6, the prefinal Chinese version of the BirthMARQ, along with its back-translated English version, was reviewed by two members of the development team to assess translation accuracy and conceptual equivalence with the original instrument. All suggestions from the instrument developers were revised accordingly.

### Phase II: psychometric evaluation

The psychometric properties assessed for the Chinese version of the BirthMARQ included content validity, construct validity, convergent validity, discriminant validity, measurement invariance, known-group validity, internal consistency, and test-retest reliability.

#### Study setting and sampling

Participants were recruited between May and August 2024 from the obstetric clinics of three tertiary-level public hospitals in Shenzhen, Guangdong Province, China, using a convenience sampling method. The eligible participants were primiparous women aged 18 to 49 years who had undergone vaginal childbirth and had an infant aged 0 to 12 months. Women who had been diagnosed with a psychiatric disorder prior to childbirth, had cognitive impairment or communication difficulties, or were currently receiving psychiatric medication or psychological therapy were excluded. The sample size for the study was planned to ensure adequate statistical power for multiple key psychometric analyses. A sensitivity power analysis was first conducted for the confirmatory factor analysis (CFA) using the semPower package in R (Moshagen & Bader, 2024). This indicated that a minimum of *N* = 120 participants was required to detect a potential model misfit (rejecting H_0_: RMSEA ≤ 0.05) with 80% power at a significance level of *α* = 0.05 for the specified model (*df* = 175). Furthermore, a key objective was to perform known-groups validity analysis, which required ensuring sufficient representation of clinical subgroups for comparison. Considering the low prevalence of conditions such as postpartum PTSD (estimated prevalence 4.7% in 17,733 samples) (Heyne et al., 2022), a target sample of approximately 500 participants was planned to enable robust statistical comparisons involving this low-prevalence group.

#### Measure

##### Demographic information questionnaire.

The demographic information questionnaire comprised seven items covering age, marital status, ethnicity, educational level, occupation, family per capita monthly income, and the time elapsed since the birth.

##### Birth memories and recall questionnaire.

The original BirthMARQ is a 21-item instrument designed to measure the characteristics of birth memories (Foley et al., 2014). The 21 items cover six domains, namely Emotional Memory (five items), Reliving (four items), Centrality of Memory (four items), Sensory Memory (four items), Recall (two items), and Coherence (two items). Each item is rated on a 7-point Likert scale (1–7), with items 1, 4, and 21 being reverse-scored. Domain scores are calculated by averaging the items within each domain. Higher scores in each domain reflect greater negative and/or mixed emotions at birth and during recalling birth (Emotional Memory), more vivid reliving of the birth experience (Reliving), a more central role of the birth memory in the mother’s sense of identity (Centrality of Memory), clearer sensory impressions (Sensory Memory), more frequent involuntary recall of the birth (Recall), and more coherent memories (Coherence). The original BirthMARQ has demonstrated satisfactory internal consistency, with Cronbach’s alpha coefficients ranging from 0.74 to 0.84 for the subscales (Foley et al., 2014). The authors have permission to use this instrument from the copyright holders.

##### Edinburgh postnatal depression scale.

The 10-item Edinburgh Postnatal Depression Scale (EPDS) was used to measure PPD (Cox, Holden & Sagovsky, 1987). It is the most widely used tool for screening PPD worldwide. Each item is rated on a 4-point scale (0–3), with total scores ranging from 0 to 30. A cut-off value of 10.5 is recommended for screening clinical depression among Chinese postpartum women (Lau et al., 2010). The Chinese version of the EPDS has demonstrated good validity and reliability, with a Cronbach’s alpha of 0.78 (Lau et al., 2010). The authors have permission to use this instrument from the copyright holders. In this study, the Cronbach’s alpha coefficient for the EPDS was 0.83.

##### Perinatal posttraumatic stress disorder questionnaire.

The Perinatal Posttraumatic Stress Disorder Questionnaire (PPQ) was utilized to measure CB-PTSD (DeMier et al., 1996). It is a 14-item self-report instrument developed according to the Diagnostic and Statistical Manual of Mental Disorders (4th Edition) criteria, comprising three subscales: arousal, avoidance, and intrusion. Respondents rate each item on a 5-point scale from 0 (not at all) to 4 (often), with total scores ranging from 0 to 56. A score of 19 or above indicates probable CB-PTSD. The Chinese version of the PPQ has demonstrated favourable validity and reliability, with a Cronbach’s alpha coefficient of 0.84 (Zhang et al., 2018). The authors have permission to use this instrument from the copyright holders. In this study, the Cronbach’s alpha coefficient for the PPQ was 0.81.

#### Data collection

Data were collected using paper-based questionnaires, and all information was obtained on-site through face-to-face interaction by a researcher who had received systematic training. Prior to administering the questionnaire, the researcher verified each participant’s identity through brief face-to-face communication and/or by reviewing relevant records in the obstetric outpatient electronic medical system, and assessed whether the individual met the predefined inclusion criteria. For women who met the eligibility criteria, the researcher provided a detailed explanation of the study purpose, survey procedures, and informed consent requirements while they were waiting for their consultation, and addressed any questions raised by the invitees. Participants were also informed about the approximate time required to complete the questionnaire, the confidentiality and anonymization procedures for data handling, and the necessity of signing written informed consent. Although real names were required on the consent form for ethical documentation, the research team assured participants that all questionnaire data would be stored and analyzed anonymously to ensure strict protection of their privacy. Completed questionnaires were returned directly to research staff and underwent on-site verification for completeness before participants were permitted to leave. Participants typically required about 10–15 min to complete the questionnaires. Additionally, we used a convenience sampling method to invite 75 participants who completed the initial assessment and agreed to participate in the follow-up evaluation. These participants completed the Chinese version of the BirthMARQ again after a 2–4 week interval to assess test–retest reliability (Yasir, 2016).

#### Statistical analysis

All statistical analyses were conducted in R version 4.4.1 (R Core Team, 2024). Demographic characteristics were summarized using descriptive statistics, where continuous variables were reported as mean and standard deviation (*SD*), and categorical variables were presented as frequency and percentage. The mean, standard deviation, and percentile ranges of each BirthMARQ subscale were calculated to describe the distribution of subscale scores.

##### Content validity.

The content validity of the Chinese version of the BirthMARQ was appraised by an expert panel. The panel comprised two psychiatrists, two psychiatric nurse specialists, one obstetrician, and two obstetric nurse specialists. Each expert rated the relevance of each item to its corresponding dimension using a 4-point Likert scale (1 = not relevant, 4 = highly relevant). The item-level content validity index (I-CVI) was calculated as the proportion of experts rating each item as 3 or 4 out of the total number of experts. The scale-level content validity index (S-CVI) was determined by averaging all I-CVI. I-CVI ≥ 0.78 and S-CVI ≥ 0.90 were deemed to indicate good content validity (Polit & Beck, 2006).

##### Construct validity.

CFA was performed using the “lavaan” package (Rosseel, 2012), with the weighted least squares with mean and variance adjustment (WLSMV) estimator, which is designed for ordinal categorical variables. The goodness-of-fit of the CFA model was appraised using the chi-square to degrees of freedom ratio (*χ*^2^/df), Comparative Fit Index (CFI), Tucker-Lewis Index (TLI), Root Mean Square Error of Approximation (RMSEA), and Standardized Root Mean Square Residual (SRMR). The model was considered acceptable and confirmable when *χ*^2^/df < 5, CFI > 0.90, TLI > 0.90, SRMR < 0.08, and RMSEA < 0.10 (Browne & Cudeck, 1992; Hu & Bentler, 1999; Kenny, Kaniskan & McCoach, 2015). Modification indices were used to identify potential sources of model misfit, and a 1,000-replication bootstrap stability analysis was performed to evaluate the robustness of the residual covariances included in the modified model. Spearman rank correlations were utilized to explore the relationships among the subscales, as well as between the time since childbirth and the six subscales.

### Convergent validity and discriminant validity

The average variance extracted (AVE) and the composite reliability (CR) were used to evaluate the convergent validity of the BirthMARQ subscales. Convergent validity was considered acceptable when AVE was ≥ 0.50 and CR was ≥ 0.70 (Fornell & Larcker, 1981). Discriminant validity was assessed using the heterotrait–monotrait ratio of correlations (HTMT), with values below 0.85 indicating adequate discriminant validity (Henseler, Ringle & Sarstedt, 2015).

### Invariance test

To assess the potential equivalence of the measurement model across different EPDS subgroups (with or without PPD symptoms), multiple-group CFA was conducted to evaluate the measurement invariance of the BirthMARQ. This evaluation sequentially examined configural, metric, and scalar invariance. Measurement invariance was considered to be supported when ΔCFI ≤ 0.010 and ΔRMSEA ≤ 0.015 (Chen, 2007).

#### Known-group validity

To evaluate known-group validity, quasi-independent variables were generated for PPD (none, probable) and CB-PTSD (none, probable). Mann–Whitney tests were employed to examine whether there were differences in subscale scores between women with and without probable PPD or CB-PTSD.

### Internal consistency, test–retest reliability and measurement error

Internal consistency of the Chinese version of the BirthMARQ and its subscales was evaluated with Cronbach’s alpha and McDonald’s omega coefficient, with a value above 0.70 indicating acceptable internal consistency (Terwee et al., 2007; McNeish, 2018). Test-retest reliability was determined using the intraclass correlation coefficient (ICC), with a value greater than 0.70 suggesting favorable reliability (Souza, Alexandre & Guirardello, 2017). Measurement error was examined using the standard error of measurement (SEM) and the smallest detectable change (SDC).

### Ethical considerations

Authorization to use the instruments for this study was obtained from the original authors. Research ethics approval was granted by the ethics committees of the three hospitals involved in the study, with each approval secured prior to the initiation of recruitment and data collection at their respective sites. The approval numbers and dates are as follows: Shenzhen Hospital, Southern Medical University (Approval Number: NYSZYYEC20230066, approved on 3 November 2023), The People’s Hospital of Baoan Shenzhen (Approval Number: BYL20231106, approved on 4 December 2023), and Shenzhen LongHua Maternity & Child Healthcare Hospital (Approval Number: SRE-PCFR/2024043, approved on 24 July 2024). All participants provided written informed consent before enrollment.

## Results

### Recruitment and participant flow

**Figure 1:**
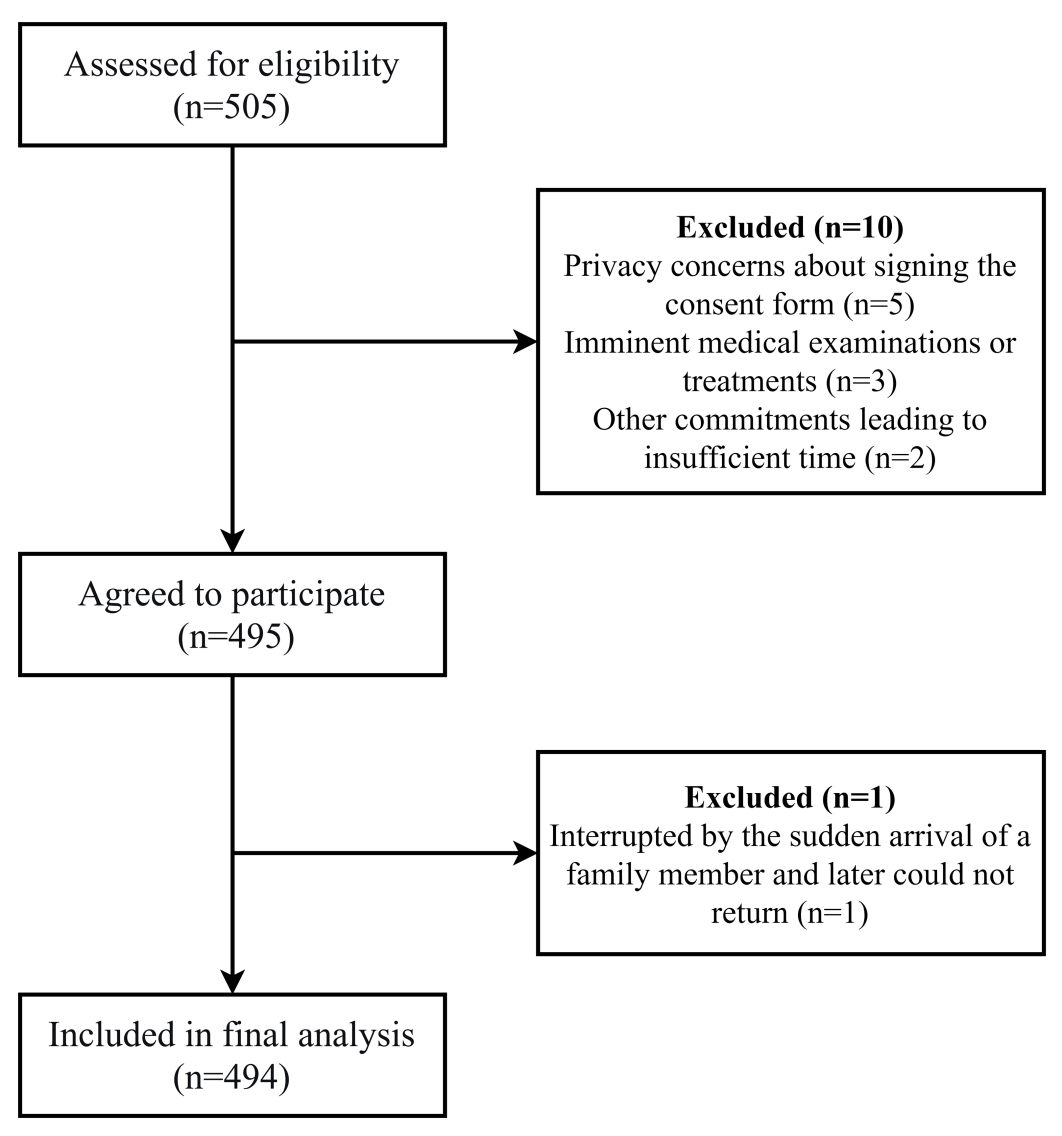
Flow diagram of participant recruitment.

A total of 505 eligible postpartum women were approached, of whom 495 agreed to participate. During recruitment, 10 participants declined participation, and one questionnaire was excluded due to incomplete responses. Ultimately, 494 participants were included in the final analysis. The recruitment flow is presented in Fig. 1.

### Characteristics of the sample

The mean age of the respondents was 29.33 years (SD = 3.12), and the average time elapsed since birth was 12.79 weeks (SD = 9.26). Most respondents were married (98.99%), of Han ethnicity (95.34%), and had a bachelor’s degree or above (71.05%). Additionally, more than half were employed in enterprises or public institutions (59.31%). Table 1 presents the detailed characteristics of the sample.

**Table 1:** Sample demographic characteristics.

Characteristic	Total (*N* = 494)
Age (years), mean (*SD*^a^), range	29.33 (3.12), 19–39
Time elapsed since the birth (weeks), mean (*SD*^a^), range	12.79 (9.26), 4–51
**Marital status, n (%)**	
Married^b^	489 (98.99%)
Single^c^	5 (1.01%)
**Ethnicity, n (%)**	
Han	471 (95.34%)
Minority	23 (4.66%)
**Highest educational level, n (%)**	
Junior college and below	143 (28.95%)
Bachelor’s degree and above	351 (71.05%)
**Occupation, n (%)**	
Unemployed	66 (13.36%)
Enterprise or Public Institution	293 (59.31%)
Self-employed	36 (7.29%)
Other	99 (20.04%)
**Family per capita monthly income (CNY^d^), n (%)**	
≤12,000	203 (41.09%)
12,001–24,000	187 (37.85%)
≥24,001	104 (21.05%)

**Notes.**

a *SD*, standard deviation.

b Includes first marriage, remarriage, and reconciliation with a previous spouse.

c Includes never married, divorced, and widowed.

d CNY, Chinese Yuan.

### Distribution of BirthMARQ subscale scores

The distribution of scores for the six BirthMARQ subscales is presented in Table S2. Emotional Memory (median = 3.20), Reliving (median = 3.00), Centrality of Memory (median = 3.75), and Sensory Memory (median = 3.25) were at typical mid-range levels. Involuntary Recall showed generally low scores (median = 2.50), whereas Coherence had the highest scores among the subscales (median = 6.50).

### Content validity

The I-CVI of the Chinese version of the BirthMARQ ranged from 0.86 to 1.00, and the S-CVI was 0.95, indicating favorable content validity.

### Construct validity

**Figure 2:**
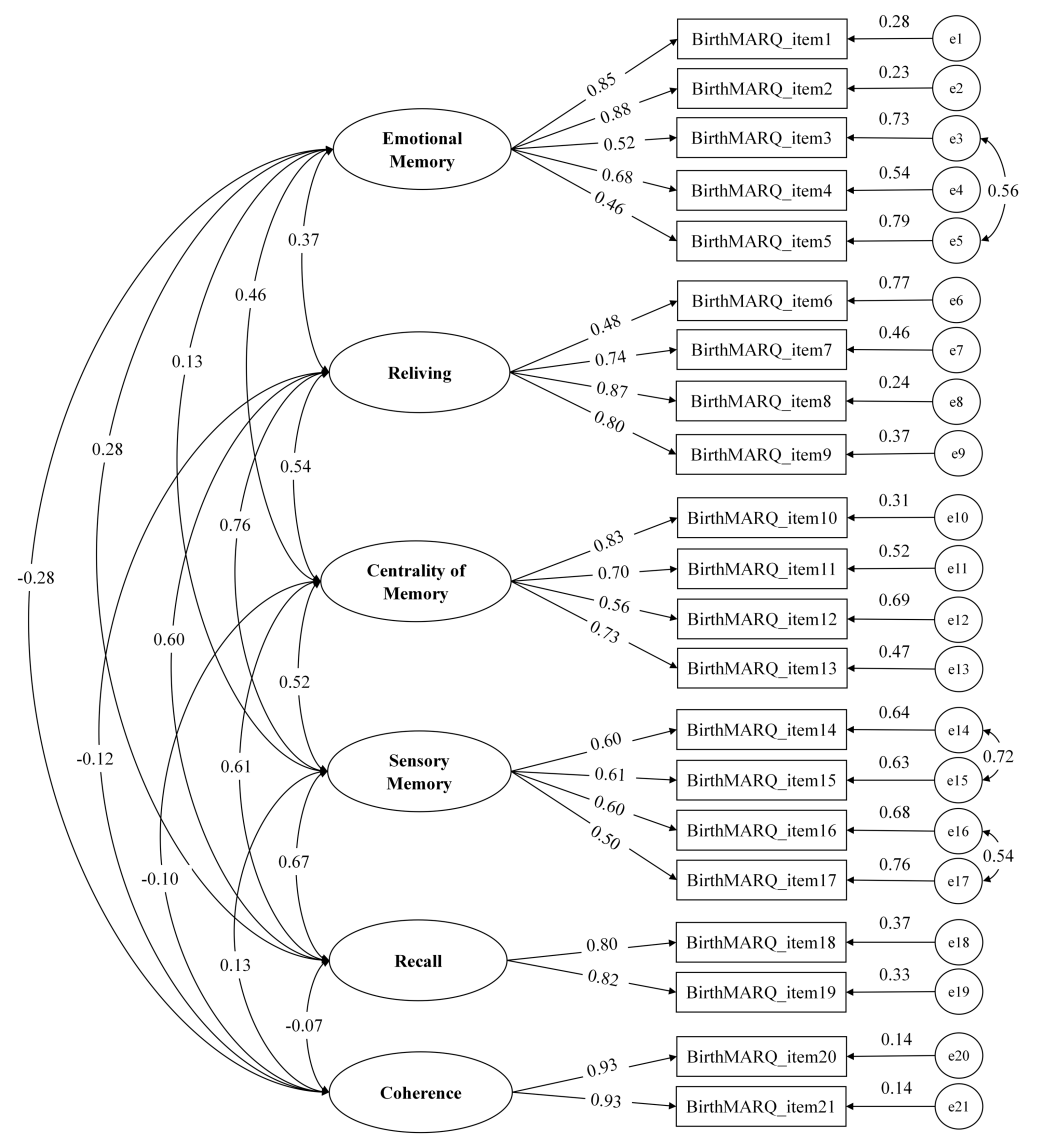
Modified confirmatory factor analysis model of the Chinese version of the BirthMARQ (Standardized Estimates). Abbreviations: BirthMARQ, Birth Memories and Recall Questionnaire.

Table 2 presents the fit indices for various models. The CFA model based on the original six-factor structure of the BirthMARQ showed a poor fit (*χ*^2^/*df* = 6.459; CFI = 0.892; TLI = 0.870; RMSEA = 0.105; SRMR = 0.087). Following inspection of the modification indices, we specified the covariance between adjacent items: items 3 and 5, 14 and 15, and 16 and 17 (Fig. 2). With these adjustments, the modified model demonstrated good fit, as indicated by all indices (*χ*^2^/*df* = 4.509, CFI = 0.932, TLI = 0.917, RMSEA = 0.084, SRMR = 0.073). The standardized factor loadings for the items ranged from 0.46 to 0.93, all significantly loading onto their respective factors, and the factor correlations were also stable, ranging from −0.28 to 0.76 (Table 3 and Fig. 2).

**Table 2:** Fit statistics for the Chinese version of BirthMARQ in the present study.

**Model**	*χ*^2^/df	CFI	TLI	RMSEA	SRMR
Initial model^a^	6.459	0.892	0.870	0.105	0.087
Modified model^b^	4.509	0.932	0.917	0.084	0.073

**Notes.**

a Original 6-factor-model with 21 items.

b Alternative 6-factor model with 3 correlated pairs of residuals: items 3 and 5, 14 and 15, and 16 and 17.

Abbreviations BirthMARQ Birth Memories and Recall Questionnaire CFI Comparative Fit Index RMSEA Root Mean Square Error of Approximation *χ*^2^/df Chi-Square to Degrees of Freedom Ratio SRMR Standardized Root Mean Square Residual TLI Tucker-Lewis Index

**Table 3:** Median scores and factor loadings for items on the Chinese version of BirthMARQ .

BirthMARQ items	Median	*IQR*	Factor loading^a^	Cronbach’s *α*	McDonald’s *ω*
Emotional Memory				0.76	0.77
1. At that time, my emotions were extremely positive^*^	3	3	0.85		
2. At that time, my emotions were extremely negative	1	3	0.88		
3. At that time, I had mixed emotions of positive and negative feelings	4	4	0.52		
4. When I recall the birth now, my emotions are extremely positive^*^	3	2	0.68		
5. When I recall the birth now, I have mixed emotions of positive and negative feelings	3	4	0.46		
Reliving				0.74	0.76
6. When I remember the birth now, I relive the visual impressions at the time	6	3	0.48		
7. When I remember the birth now, I relive the bodily sensations at the time	3	3	0.74		
8. When I remember the birth now, I feel like I’m reliving it, as if it’s currently happening rather than something in the past	2	2	0.87		
9. When I remember the birth now, I relive the sound(s) at the time	2	3	0.80		
Centrality of Memory				0.75	0.75
10. The experience of birth has affected the way I think and feel about other experiences	3	4	0.83		
11. The experience of birth has become central to my understanding of myself and the world	3	4	070		
12. The experience of birth was a turning point in my life	5	3	0.56		
13. I often think about the impact the experience of birth will have on my future	3	3	0.73		
Sensory Memory				0.73	0.74
14. When I recall the birth, I can remember the smells at the time	1	1	0.60		
15. When I recall the birth, I can remember the tastes at the time	1	1	0.61		
16. When I recall the birth, I can remember the sounds at the time	5	2	0.60		
17. When I recall the birth, I can remember the touches at the time	5	3	0.50		
Recall				0.74	0.74
18. My memory (or parts of the memory) of the birth will suddenly pop out of nowhere without me consciously thinking about it	2	3	0.80		
19. What is currently happening will unexpectedly bring back my memory (or parts of the memory) of the birth	3	3	0.82		
Coherence				0.89	0.89
20. My memory of the birth is logical and coherent, with no major gaps	6	2	0.93		
21. My memory of the birth is fragmented, which means it’s scattered and partially missing^*^	7	1	0.93		

**Notes.**

a Standardized factor loadings in the modified confirmatory factor analysis model of the Chinese version of BirthMARQ.

* Indicates the item should be reverse scored.

Abbreviations BirthMARQ Birth Memories and Recall Questionnaire IQR Interquartile Range

Bootstrap analysis results indicated that all three added residual covariances demonstrated stable estimates, with positive 95% bootstrap confidence intervals: items 3 and 5 (0.33–0.52), items 14 and 15 (0.31–0.57), and items 16 and 17 (0.26–0.50).

### Correlations between subscales

Table 4 shows the correlations between the six subscales. Although many subscales were significantly correlated, most coefficients exhibited small effect sizes. The strongest relationships, with medium effect sizes (*r* = 0.30 to 0.45), were positive associations between Emotional Memory and Centrality of Memory, as well as positive interrelationships among Reliving, Centrality of Memory, Sensory Memory, and Involuntary Recall. Notably, the length of time since birth was not significantly correlated with any of the subscales except Reliving.

**Table 4:** Spearman correlation coefffcient between subscales of the Chinese version of BirthMARQ and time since birth.

Component	1	2	3	4	5	6
1. Emotional memory						
2. Reliving	0.27^***^					
3. Centrality of memory	0.34^***^	0.38^***^				
4. Sensory memory	0.09^*^	0.44^***^	0.30^***^			
5. Involuntary recall	0.21^***^	0.41^***^	0.45^***^	0.41^***^		
6. Coherence	−0.19^***^	−0.09	−0.08	0.10^*^	−0.10^*^	
7. Time since birth (weeks)	0.01	−0.12^*^	0.05	−0.09	−0.01	0.05

**Notes.**

* *P* < 0.05.

*** *P* < 0.001.

Abbreviations BirthMARQ Birth Memories and Recall Questionnaire

### Convergent validity and discriminant validity

The AVE and CR for the six subscales are presented in Table 5. Except for the Sensory Memory subscale, all other subscales demonstrated acceptable convergent validity. The HTMT ranged from 0.029 to 0.622, indicating good discriminant validity among the six factors.

**Table 5:** Convergent validity of the Chinese version of BirthMARQ.

	Emotional memory	Reliving	Centrality of memory	Sensory memory	Recall	Coherence
AVE	0.49	0.54	0.51	0.33	0.65	0.86
CR	0.72	0.77	0.77	0.47	0.75	0.89

**Notes.**

Abbreviations BirthMARQ Birth Memories and Recall Questionnaire AVE Average Variance Extracted CR Composite Reliability

### Invariance test

Measurement invariance across EPDS subgroups was examined using a series of nested models (Table 6). For metric invariance, the changes in model fit indices were small (ΔCFI = −0.005; ΔRMSEA = 0.001). In addition, the test for scalar invariance showed no significant decrease in model fit (ΔCFI = 0.001; ΔRMSEA = 0.000), indicating that the Chinese version of the BirthMARQ achieved scalar invariance across the EPDS groups.

**Table 6:** Test of measurement invariance across EPDS groups.

Model	*χ* ^2^	df	CFI	RMSEA (90% CI)	△CFI	△RMSEA
Configura invariance	849.057	344	0.934	0.077 (0.071–0.084)		
Metric invariance	898.421	358	0.929	0.078 (0.072-0.085)	−0.005	0.001
Scalar invariance	887.873	352	0.930	0.079 (0.072-0.085)	0.001	0.000

**Notes.**

Abbreviations EPDS Edinburgh Postnatal Depression Scale *χ*2 Satorra-Bentler chi-square df degrees of freedom CFI Comparative Fit Index RMSEA Root Mean Square Error of Approximation Δ CFI change in CFI compared with the previous model Δ RMSEA change in RMSEA compared with the previous model

### Known-group validity

In the present study, 18% of women (*n* = 87) were identified with probable PPD, while 8% (*n* = 41) met the criteria for probable CB-PTSD. Most women with probable CB-PTSD also exhibited probable PPD (*n* = 30). Table 7 presents descriptive and inferential statistics for all subscales of the Chinese version of the BirthMARQ, categorized by PPD and CB-PTSD.

**Table 7:** Median scores of the Chinese version of BirthMARQ subscales by PPD and CB-PTSD.

		PPD		*P* value	CB-PTSD		*P* value
	**Overall** (*N*= 494)	None (*N*= 407)	Probable (*N*= 87)		None (*N*= 453)	Probable (*N*= 41)	
Emotional memory	3.20	3.00	3.80	<0.001	3.00	4.40	<0.001
Reliving	3.00	3.00	3.50	<0.001	3.00	4.25	<0.001
Centrality of memory	3.75	3.75	4.50	<0.001	3.75	5.25	<0.001
Sensory memory	3.25	3.25	3.25	0.223	3.25	4.00	0.003
Involuntary recall	2.50	2.50	3.50	<0.001	2.50	4.50	<0.001
Coherence	6.50	6.50	6.00	<0.001	6.50	6.00	0.700

**Notes.**

Abbreviations BirthMARQ Birth Memories and Recall Questionnaire PPD postpartum depression CB-PTSD Childbirth-related post-traumatic stress disorder

The results revealed differences between women with and without probable PPD and CB-PTSD. Women with probable PPD scored higher on Emotional Memory, Reliving, Centrality of Memory, and Involuntary Recall (all *P* < 0.001), and lower on Coherence (*P* < 0.001) than those without probable PPD. No significant difference was found for Sensory Memory (*P* = 0.223). Women with probable CB-PTSD scored higher on Emotional Memory, Reliving, Centrality of Memory, Sensory Memory, and Involuntary Recall (all *P* < 0.05) compared to women without probable CB-PTSD. No significant difference was observed for Coherence (*P* = 0.700).

### Internal Consistency, test–retest reliability and measurement error

The Cronbach’s alpha coefficient for the Chinese version of the BirthMARQ was 0.83 and McDonald’s omega coefficient was 0.81. The subscale coefficients ranged from 0.73 to 0.89 for Cronbach’s alpha and from 0.74 to 0.89 for McDonald’s omega across subscales (Table 3). Over a mean interval of 18.44 days (SD = 3.45, range = 14–28 days), test-retest reliability was assessed using a two-way random-effects model for absolute agreement (ICC[2,1]). The ICC for the total scale was 0.73 (95% CI [0.56–0.84]). The SEM was 0.37, and SDC was 1.01. These results showed that the Chinese version of the BirthMARQ had an acceptable internal consistency, test-retest reliability and measurement precision.

## Discussion

This study systematically translated and cross-culturally adapted the BirthMARQ into Chinese with the widely used Beaton model (Beaton et al., 2000). A multicenter cross-sectional survey was conducted among Chinese postpartum women to verify the psychometric properties of the questionnaire. The results indicated that the Chinese version of the BirthMARQ demonstrated acceptable validity and reliability. Thus, it can be used as an effective tool to assess birth memory characteristics in postpartum women and to identify individuals with negative or traumatic birth memories across multiple domains.

The CFA supported the six-factor structure of the original BirthMARQ, with three pairs of significant covariances identified. The covariance between item 3 (*At that time, I had mixed emotions of positive and negative feelings*) and item 5 (*When I recall the birth now, I have mixed emotions of positive and negative feelings*) can be explained by mood-congruent memory, a psychological phenomenon in which emotional memory is biased toward content affectively congruent with a past or current mood (Faul & LaBar, 2023). The covariance between item 14 (*When I recall the birth, I can remember the smells at the time*) and item 15 (*When I recall the birth, I can remember the tastes at the time*) may stem from the neural integration of taste and smell in the brain (Small et al., 2004) which creates a unified sensory representation and facilitates the simultaneous recall of these memories. Similarly, the covariance between item 16 (*When I recall the birth, I can remember the sounds at the time*) and item 17 (*When I recall the birth, I can remember the touches at the time*) may arise from the multisensory interactions between the auditory and somatosensory systems, whereby sound enhances the processing of touch (Kayser et al., 2005; Ro, Ellmore & Beauchamp, 2013).

In terms of convergent validity, the Sensory Memory factor showed relatively weaker convergence. Prior studies have also noted that sensory impressions such as smell, taste, and tactile sensations may vary considerably across individuals (Foley et al., 2014; Topkara & Çağan, 2021). Given the inherently subjective and context-dependent nature of sensory experiences, such variability may partially account for the weaker convergence observed in this dimension. Future research may further investigate the specific factors that contribute to the variability of sensory-based childbirth recollections.

Analyses of differences in birth memory characteristics between women with probable PPD and CB-PTSD and those without demonstrated that the Chinese version of the BirthMARQ has good known-group validity. In this study, women with probable PPD reported stronger negative and/or mixed emotional memories, more frequent reliving and involuntary recall, and memories that were more central to their identities, but showed no significant differences in sensory memory compared with women without probable PPD, consistent with previous research (Newby & Moulds, 2011; Foley et al., 2014; Parry & O’Kearney, 2014; Payne et al., 2019). However, it is noteworthy that women with probable PPD reported their memories of childbirth to be less complete and coherent. This finding aligns with research indicating a negative correlation between memory coherence and depressive symptoms (Vanderveren, Bijttebier & Hermans, 2019), but contrasts with Foley et al. (2014), who found no significant differences in coherence between depressed and non-depressed women. This discrepancy may result from the latter study including multiparous and cesarean section women, while our study focused solely on primiparous women, as well as possible cultural influences. Future research could further explore this relationship while controlling for potential confounding factors (*e.g.*, obstetric variables) to clarify the specific influences on birth memory coherence and PPD.

Women with probable CB-PTSD reported more mixed and/or negative memories, memories more central to their identities, and more involuntary recall compared to those without probable CB-PTSD. These findings are consistent with previous research (Ayers, 2007; Foley et al., 2014; Thiel et al., 2021) and support established theoretical frameworks of PTSD. Specifically, the finding that birth memories are more central to the identity of women with probable CB-PTSD supports (Berntsen, Willert & Rubin, 2003) landmark hypothesis, which posits that traumatic memories in PTSD serve as crucial reference points for organizing other personal memories. High involuntary recall ratings from women with probable CB-PTSD are consistent with Ehlers & Clark’s (2000) cognitive model, which proposes that trauma memories are more prone to automatic cueing. Moreover, women with probable CB-PTSD reported birth memories containing more sensory details and experienced more frequent reliving of the birth experience, which aligns with the findings of Thiel et al. (2021) but contrasts with those of Foley et al. (2014), who found that women with probable CB-PTSD did not report greater reliving and reported fewer sensory impressions than those without. This discrepancy may be partly attributed to differences in sample characteristics and may indirectly reflect the potential moderating role of cultural factors in the processing of birth memories, underscoring the need for further research and cross-cultural exploration.

Interestingly, our study found no significant association between the coherence of birth memories and CB-PTSD, which contrasts with previous findings (Foley et al., 2014; Ayers, Radoš & Balouch, 2015; Thiel et al., 2021). Research based on self-report assessments has shown that women with probable CB-PTSD describe their birth memories as less coherent (Foley et al., 2014; Thiel et al., 2021). However, studies using narrative methods have yielded opposite results, indicating that women with probable CB-PTSD demonstrated greater coherence when recounting their traumatic childbirth experiences (Ayers, Radoš & Balouch, 2015). This inconsistency can be explained by the dual representation theory of PTSD (Brewin, Dalgleish & Joseph, 1996), which posits that traumatic memories can be stored both as verbally accessible memories (organized and coherent) and as situationally accessible memories (fragmented and sensory-based). Self-reported coherence measures may reflect individuals’ meta-cognitive evaluations of their memories rather than the actual structural organization. Overall, the observed differences in birth memory characteristics between women grouped by probable PPD and CB-PTSD are largely consistent with existing theories and literature, supporting the good known-group validity of the Chinese version of the BirthMARQ. However, the inconsistencies require further research and exploration.

The Chinese version of the BirthMARQ demonstrated acceptable internal consistency across all factors and the overall scale, indicating that the instrument can reliably reflect postpartum women’s birth memory characteristics by computing separate scores for each factor. Furthermore, this study extended the validation of the instrument by assessing its test-retest reliability, an aspect not previously addressed. The high test-retest reliability indicates that birth memory characteristics remain stable and consistent during the postpartum period. Collectively, these findings provide robust evidence for the reliability and practical applicability of the Chinese BirthMARQ in clinical evaluation and research contexts.

### Strengths and limitations

This study’s strength lies in the rigorous translation, cultural adaptation, and psychometric validation of the BirthMARQ within the Chinese context, producing a comprehensive and user-friendly instrument that assesses birth memory characteristics to support future research and clinical practice. However, several limitations should be considered when interpreting the reported findings. First, this study included only primiparous women who had undergone vaginal childbirth within the first postpartum year. In addition, participants were recruited from three tertiary hospitals in Shenzhen, an economically developed urban area in South China; therefore, the sample may not fully represent postpartum women from other regions or socioeconomic backgrounds in China, which may limit the generalizability of the findings. Future research should aim to include a more diverse sample, incorporating women from different regions and backgrounds, as well as those with different modes of birth, varying parity, and at different postpartum stages, to enhance the representativeness and applicability of the findings. Furthermore, although the current study centered on women’s birth memories, the instrument could be adapted to evaluate men’s birth memories, provided it undergoes appropriate validation for this purpose. Second, psychological symptoms were assessed through self-report questionnaires, which can only indicate the potential likelihood of PPD and CB-PTSD. To further validate these findings, replication with a clinical sample of women formally diagnosed with PPD or CB-PTSD would be beneficial. Third, this study employed traditional fixed fit index cutoffs in the CFA. However, these thresholds may not be suitable in all analytical contexts and can sometimes lead to overly favorable assessments of model fit (Moshagen & Bader, 2024). Future research may consider re-evaluating model fit using more advanced approaches, such as the more flexible Dynamic Fit Index cutoff method (Moshagen & Bader, 2024). Finally, although this study provides evidence for the psychometric robustness of the Chinese BirthMARQ, further exploration of cultural factors uniquely influencing birth memories among Chinese women is warranted.

### Research and practical implications

The subscale scores of the Chinese BirthMARQ can provide healthcare providers with a valuable reference for identifying specific birth memory characteristics that may merit further psychosocial assessment. Higher scores on Emotional Memory, Reliving, Sensory Memory, or Involuntary Recall may reflect the presence of negative or intrusive birth memories, whereas lower Coherence scores may indicate more fragmented or weakly organized recollections. These quantitative indicators can help clinicians determine whether mothers may benefit from additional postpartum counseling, psychological support, or early intervention. It is important to emphasize that the BirthMARQ is intended to facilitate early risk identification rather than to serve as a diagnostic instrument.

Future studies may employ this instrument in cross-sectional or longitudinal designs to examine associations and underlying mechanisms linking birth memory characteristics with postpartum psychological disorders (*e.g.*, PPD, CB-PTSD, anxiety), as well as the impact of prenatal, intrapartum, and postpartum factors on birth memory. Researchers can design intervention programs and conduct randomized controlled trials or quasi-experimental studies to investigate effective strategies for intervening in negative or traumatic birth memories to prevent and control the onset and progression of postpartum psychological disorders. In clinical practice, this questionnaire can serve as a practical and effective screening tool to help healthcare professionals identify mothers who may exhibit negative or traumatic birth memory characteristics. Based on assessment findings, personalized psychological interventions (*e.g.*, midwife-led birth debriefing, trauma-informed supportive counseling, cognitive behavioral therapy, guided expressive writing, mindfulness-based interventions, or brief psychoeducation) can be designed and implemented to facilitate the restructuring of such memories, thereby reducing the risk of associated postpartum psychological disorders.

## Conclusion

The validation of the Chinese version of the BirthMARQ provides a valid and reliable instrument for assessing birth memory characteristics among postpartum women in China, addressing a critical gap in the field. It is a valuable instrument for future research and clinical practice, enabling healthcare professionals to identify women with negative or traumatic birth memories and develop individualized interventions based on these memory characteristics to reduce the risk of associated postpartum psychological disorders. However, a highly representative sample is necessary to confirm the psychometric properties and clinical effectiveness of the Chinese version of the questionnaire.

##  Supplemental Information

10.7717/peerj.20814/supp-1 Supplemental Information 1 STROBE Checklist

10.7717/peerj.20814/supp-2 Supplemental Information 2 Percentile values and distribution characteristics of the six BirthMARQ subscale scores

10.7717/peerj.20814/supp-3 Supplemental Information 3 Overall population data

10.7717/peerj.20814/supp-4 Supplemental Information 4 Test–retest reliability data

10.7717/peerj.20814/supp-5 Supplemental Information 5 Codebook of categorical data for all variables in the raw data

## Additional Information and Declarations

### Data Availability

The following information was supplied regarding data availability: The raw measurements are available in the Supplemental Files.
