# Supplementary material for: Cross-cultural validation of the birth memories and recall questionnaire: a cross-sectional study among Chinese postpartum women
Source: PeerJ. 2026 Feb 26;14:e20814. doi: 10.7717/peerj.20814 (PMC12950182; doi:10.7717/peerj.20814)
Supplement: Supplemental Information 2 [file peerj-14-20814-s002.docx]

**Supplementary Table 1.**

**Percentile values and distribution characteristics of the six BirthMARQ subscale scores.**

| Subscale | Mean | Standard deviation | P10 | P25 | P50 | P75 | P90 |
| --- | --- | --- | --- | --- | --- | --- | --- |
| **Emotional Memory** | 3.14 | 1.33 | 1.20 | 2.20 | 3.20 | 4.00 | 5.00 |
| **Reliving** | 3.26 | 1.30 | 1.75 | 2.50 | 3.00 | 4.00 | 5.00 |
| **Centrality of Memory** | 3.77 | 1.56 | 1.75 | 2.50 | 3.75 | 5.00 | 5.75 |
| **Sensory Memory** | 3.34 | 1.32 | 1.75 | 2.50 | 3.25 | 4.00 | 5.25 |
| **Involuntary Recall** | 2.99 | 1.63 | 1.00 | 1.50 | 2.50 | 4.00 | 5.50 |
| **Coherence** | 5.74 | 1.56 | 3.00 | 5.50 | 6.50 | 7.00 | 7.00 |
