# Supplementary material for: Cross-cultural validation of the birth memories and recall questionnaire: a cross-sectional study among Chinese postpartum women
Source: PeerJ. 2026 Feb 26;14:e20814. doi: 10.7717/peerj.20814 (PMC12950182; doi:10.7717/peerj.20814)
Supplement: Supplemental Information 5 [file peerj-14-20814-s005.docx]

**Codebook of categorical data for all variables in the raw data**

| **Variable** | **Assignment method** |
| --- | --- |
| Independent variable |  |
| race | Han =1  Minority =2 |
| occupation | Unemployed =1  Enterprise or Public Institution =2  Self-employed =3  Other =4 |
| marital.status | Married =1  Single =2 |
| educational.level | Junior college and below =1  Bachelor's degree and above =2 |
| family.per.capita.monthly.income | ≤12000 =1  12001-24000 =2  ≥24001 =3 |
| PPQ.F | Yes =1  No =0 |
| EPDS.F | Yes =1  No =0 |
